# Correlation of Central Corneal Thickness and Keratometry with Refraction and Axial Length: A Prospective Analytic Study

**DOI:** 10.7759/cureus.3917

**Published:** 2019-01-19

**Authors:** Vallinayagam Muthu Krishnan, Krishnamoorthy Jayalatha, Chellappa Vijayakumar

**Affiliations:** 1 Ophthalmology, Mahatma Gandhi Medical College and Research Institute, Puducherry, IND; 2 Ophthalmology, Dr. Agarwal's Eye Hospital, Puducherry, IND; 3 Surgery, Jawaharlal Institute of Postgraduate Medical Education and Research, Puducherry, IND

**Keywords:** axial length, corneal curvature, central corneal thickness, myopic refraction

## Abstract

Introduction

The correlation between corneal curvature and central corneal thickness (CCT), with ocular parameters like axial length and refraction, remains elusive. The various ocular biometric parameters are interdependent and their correlation varies with the population studied. A comprehensive study is required for a better understanding of the ocular biometric properties of Indian eyes.

Methodology

This is a prospective study done in 156 subjects. Subjects with clear lens and clear cornea were included in the study. Those with corneal opacity, cataract, pregnancy, and diabetes were excluded. Cycloplegic refraction, autokeratometry (Potex, ultrasonic auto keratometer), central corneal thickness (ultrasonic pachymeter), and axial length (Sonomed) were done in all subjects. Subjects were divided into two groups based on refraction, for an analysis of parameters. Group one included subjects with refraction of +3 diopters (D) to -2.99D and group two with subjects with > -3D refraction.

Results

With an increasing axial length and myopic refraction, the corneal curvature tends to be steeper. The average CCT of subjects in group one and group two were 530.34 microns and 542.63 microns, respectively. Subjects with refraction more than 10 diopters or axial length more than 25 mm had a mean CCT of 525.25 microns. Subjects with myopic refraction between 3 diopters and 10 diopters had a mean CCT of 551.32 microns.

Conclusion

Increase in corneal power is associated with increasing myopic refraction. Steeper corneal curvature is correlated with increasing axial length and thinner corneas. The mean CCT was 533.87 microns with a standard deviation (SD) of 40.02. Axial myopia is associated with an increase in corneal thickness. These ocular biometric findings have crucial implications in refractive surgeries.

## Introduction

With the recent surge in corneal refractive surgeries, there is a renewed interest in understanding the correlation between corneal curvature and central corneal thickness (CCT) with other ocular biometric parameters such as axial length (AL) and refraction [1]. The various ocular biometric parameters are interdependent. In spite of innumerable studies, the correlation remains elusive, as the results are quite variable. The correlation between different parameters varies with the population studied. There is a need to vividly study our population with a broader perspective, for a better understanding of the ocular biometric properties of Indian eyes. Hence, a prospective study was undertaken to correlate the association of CCT, corneal curvature, and AL with refractive error.

## Materials and methods

This prospective study was carried out over a period of one year in a tertiary care center in South India. This was done to correlate the association of CCL, corneal curvature, and AL with refractive error. One hundred fifty-six subjects, with an average age of 29.27 years standard deviation (SD) with mean refraction of -3.10 diopters (D), were included. There were 93 females and 65 males in the study with a mean age of 30.91 (SD 11.5) and 26.77 (SD 10.84), respectively. Subjects with clear lens and clear cornea were included in the study. Subjects with contact lenses, corneal opacity, cataract, pregnancy, diabetes, and prior ocular surgery were excluded.

Institutional human ethics committee (IEC) approval was obtained for the study. The nature, methodology, and risks involved in the study were explained to the patient and informed consent was obtained. The information collected was kept confidential and the patient was given full freedom to withdraw at any point during the study. All provisions of the Declaration of Helsinki were followed in this study. Cycloplegic refraction (with cyclopentolate), autokeratometry (Potex, ultrasonic auto keratometer), CCT (ultrasonic pachymeter), and AL (Sonomed Escalon, NY, US) were performed for all subjects. Subjects were divided into two groups based on refraction, for an analysis of parameters. Group one included subjects with refraction of +1.75D to -2.99D (n=102) and group two with subjects > -3D (n=54) refraction. For the analysis, the average values of the right and left eyes were taken. A descriptive analysis and Pearson’s bivariate co-relation of studied ocular parameters was done using the Statistical Package for the Social Sciences (SPSS) software (IBM Corp, Armonk, NY, US).

## Results

 A summary of spherical equivalent (SE in D), base curve (BC in mm), CCT in microns, and AL in mm is shown in Table 1.

**Table 1:** Summary of spherical equivalent, base curve, central corneal thickness, and axial length in study patients _★ Average; SE – Spherical Equivalent in diopters; BC – Base Curve in millimeters; CCT – Central Corneal Thickness in micrometers; AL – Axial Length in millimeters_

Group	SE (D)^_★_^	BC (mm)^_★_^	CCT (µm)^_★_^	AL (mm)^_★_^
I (n=102)	-1.30 (0.88)	7.57 (0.30)	529.33 (39.29)	22.77 (0.81)
II (n=54)	-6.49 (3.64)	7.56 (0.28)	542.44 (40.34)	24.93 (1.62)
Total (n=156)	-3.10 (3.34)	7.57 (0.29)	533.87 (40.02)	23.52 (1.54)

The average BC values and CCT values showed a normal distribution whereas the values of SE and AL were skewed to the left. The correlation was statistically significant between the BC and AL (r=0.28; p=0.000). The eyes with longer AL tend to have flatter cornea (Table 2). 

**Table 2:** Correlation of central corneal thickness and base curve with spherical equivalent and axial length in study patients _★__Average; SE – Spherical Equivalent in diopters; BC – Base Curve in millimeters; CCT – Central Corneal Thickness in micrometers; AL – Axial Length in millimeters_

Parameters	CCT^_★_^	BC^_★_^	SE^_★_^	AL^_★_^
CCT		r = 0.269; p =0.001	r = -0.119; p=0.140	r = 0.211; p=0.008
BC	r = 0.269; p =0.001		r=0.070; p=0.383	r =0.287; p=0.000

Correlations

Keratometry and Axial Length

**Figure 1:**
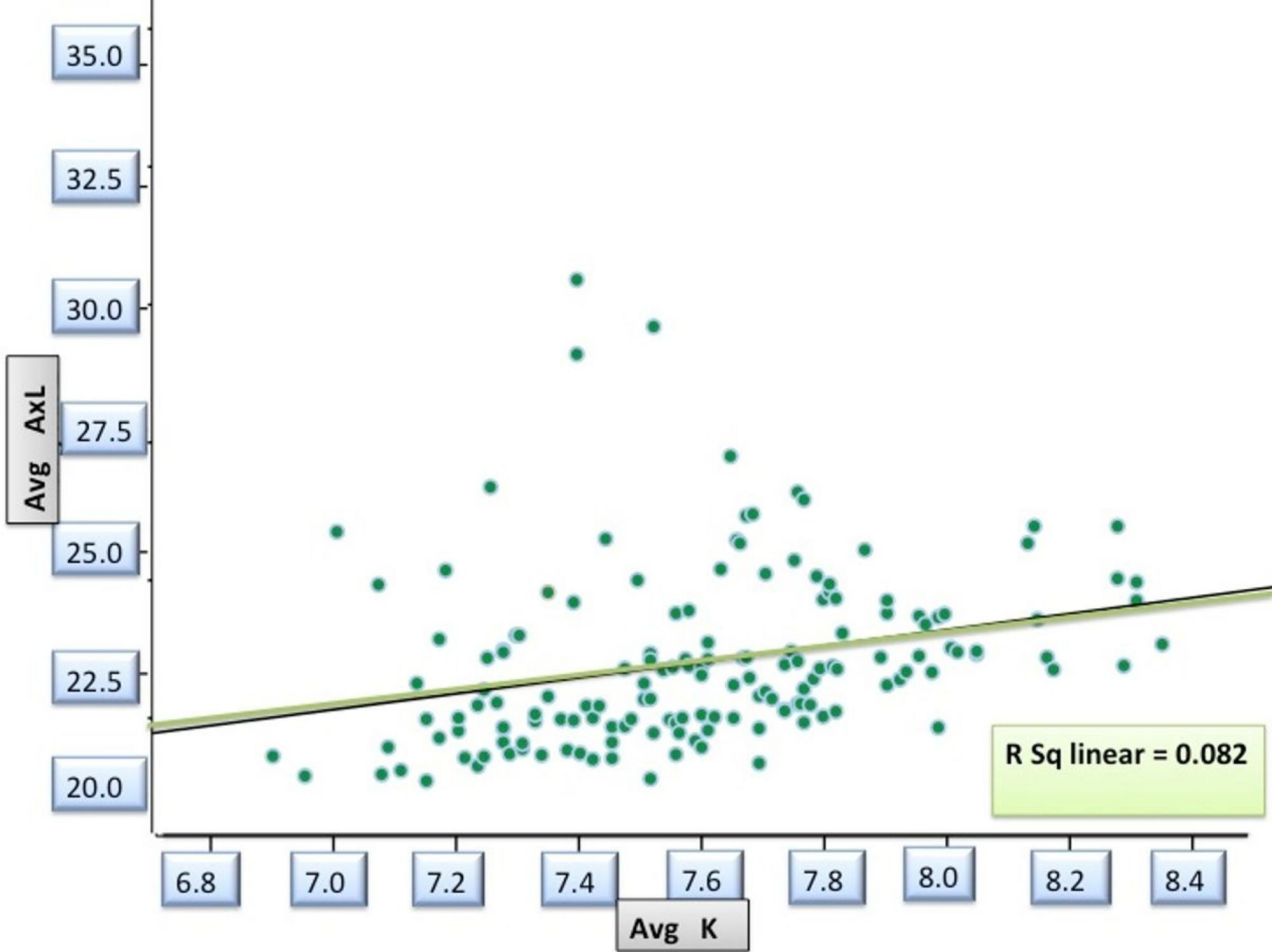
Correlation between base curve and axial length in study patients ^avg K - Averaged Corneal curvature in millimeters by Keratometry; avg AxL- Axial length in millimeters^

With the increase in AL, the cornea had a tendency to become flatter, as revealed by the keratometric values (r=0.287; p=0.00), which was statistically significant (Figure 1).

Refraction and Axial Length

**Figure 2:**
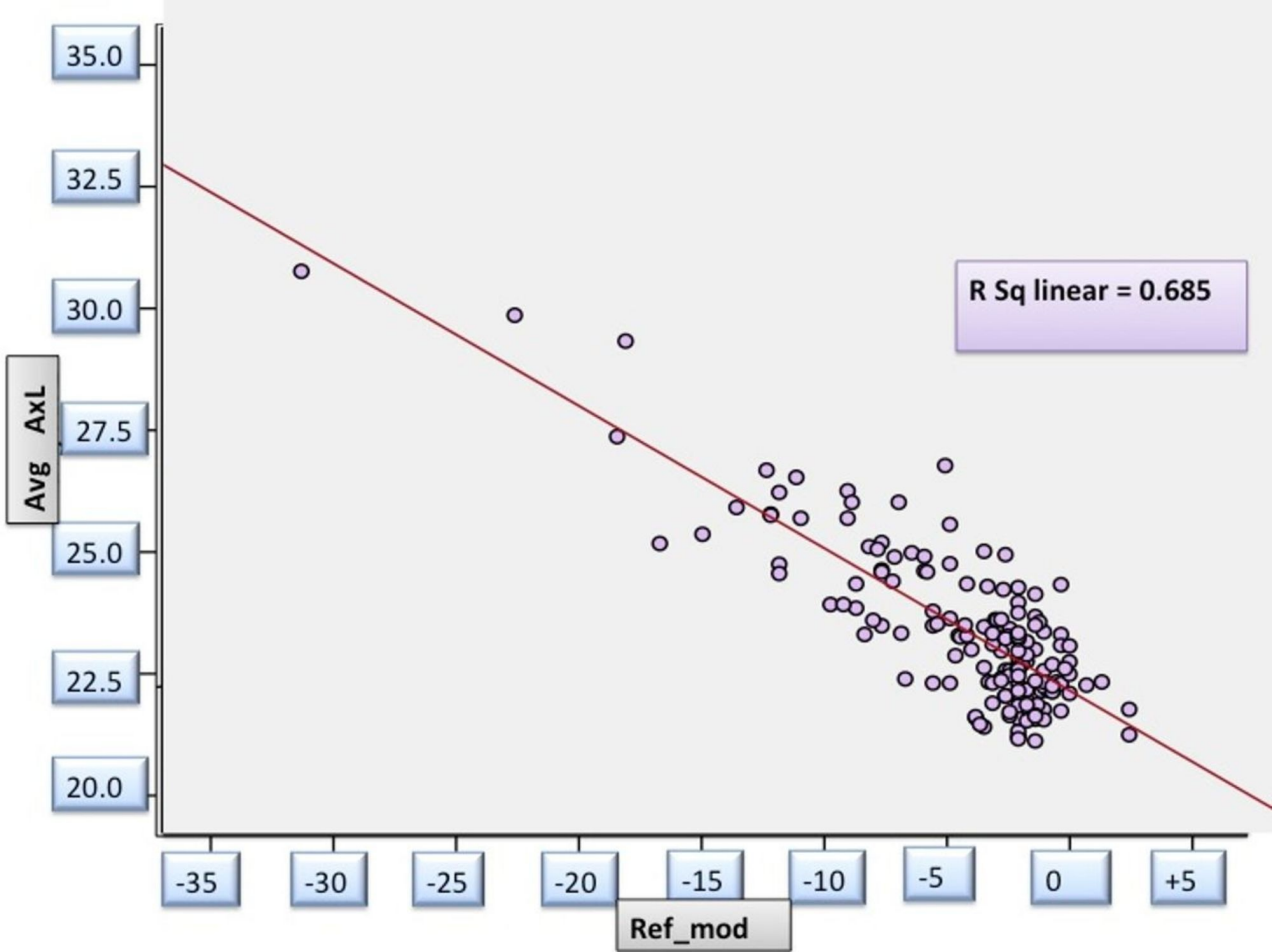
Correlation between spherical equivalent and axial length in study patients _avg AxL: Averaged Axial length in millimeters; ref_mod: Refractive error/ spherical equivalent in diopters_

With AL, the spherical equivalent had a negative correlation, i.e., with increasing myopic refraction, there was an increase in AL (r= -0.827; p= 0.00). The correlation between these two parameters was highly significant statistically (Figure 2).

Keratometry and Corneal Thickness

**Figure 3:**
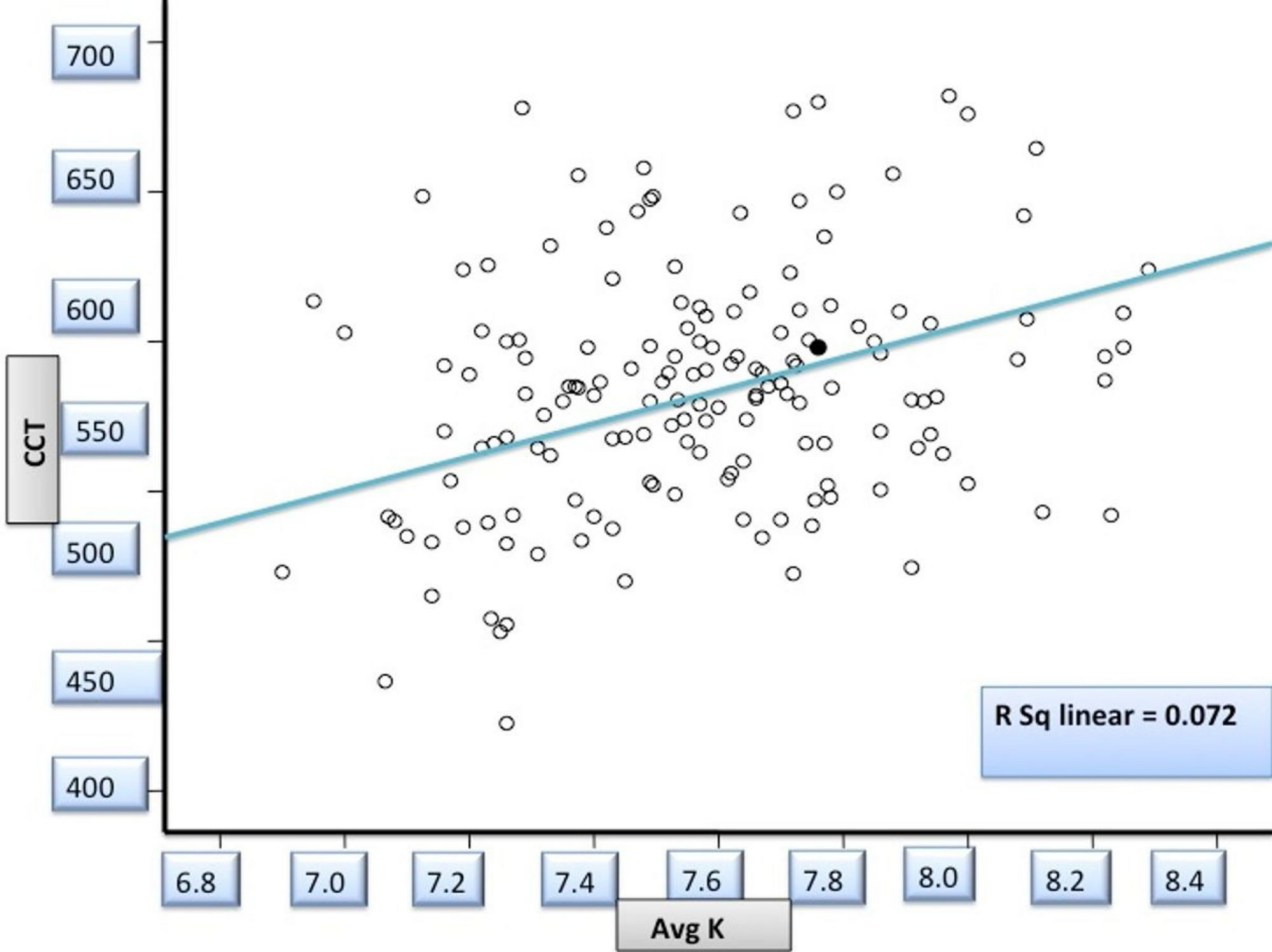
Correlation between base curve and central corneal thickness in study patients _Avg K: Averaged corneal curvature in diopters by keratometry; Avg CCT: Averaged central corneal thickness in millimeters_

With increasing BC (flattening) of the cornea, there was an increase in CCT in our study and was statistically significant (r=0.269; p=0.001) (Figure 3).

Refraction and Corneal Thickness

**Figure 4:**
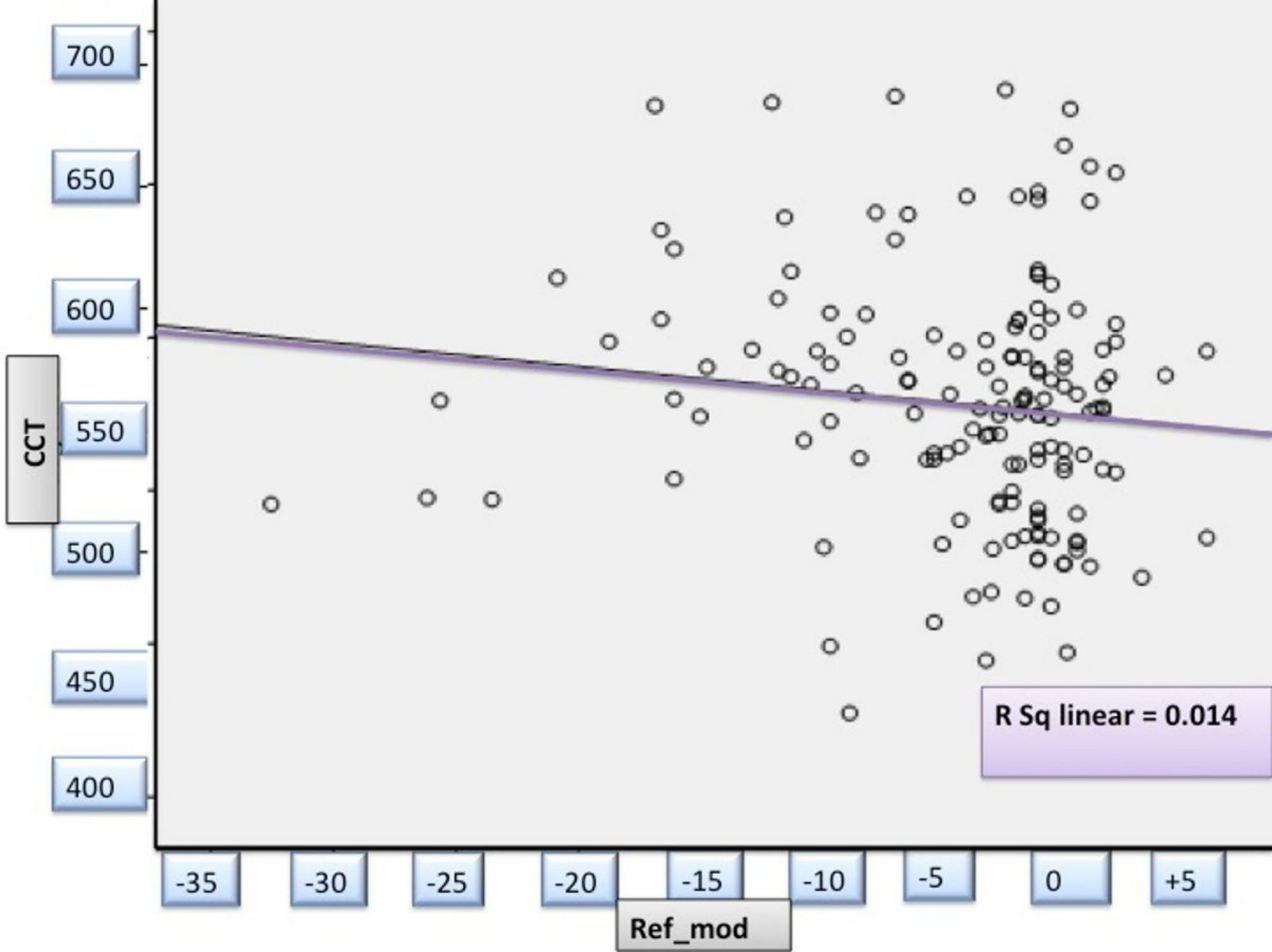
Correlation between spherical equivalent and central corneal thickness in study patients _avg CCT: Averaged Central Corneal thickness in millimeters; ref_mod: Averaged refractive errors/spherical equivalent in diopters_

With increasing myopic refraction, the CCT showed a tendency to become thicker. The correlation was statistically significant (r= -0.172; p=0.03) (Figure 4).

Refraction and Keratometry 

The SE of subjects showed a positive correlation with BC, which was not statistically significant (r=0.07; p=0.38). This suggests a tendency for the cornea to become steeper with increasing myopic refraction.

Corneal Thickness and Axial Length

The CCT showed a statistically significant positive correlation with increasing AL of the eyeball (r= 0.211; p=0.008). It indicates that eyes with increasing AL tend to have thicker cornea.

## Discussion

A comprehensive study of our population appears to be the need of the hour, for a better understanding of the ocular biometric properties of Indian eyes. The results were analyzed and discussed as follows.

The SE of refraction correlates well with AL and this positive correlation has been proven in many studies [1-2]. The present study also confirms the strong positive correlation between AL and refraction. Our study showed that with increasing myopic refraction, the cornea tends to become steeper [1,3-4]. The correlation was statistically significant between the BC and AL (r=0.28; p=0.000). The eyes with longer AL tend to have flatter cornea.

A similar association of myopic eyes and steeper corneas has been reported. A contradicting finding of a flatter cornea with increasing AL has also been reported. A subject in whom the myopia is due to the elongation of the eyeball in the initial period of ocular growth in childhood is associated with a flatter cornea. In those with adult-onset myopia, the posterior segment enlargement does not affect the anterior segment structures and are, hence, often associated with a steeper cornea. The first mechanism is Van Alphen’s “size factor” and the latter reasoning is Scott and Grosvenor’s “stretch factor” hypothesis [1,3-4]. It was proposed that a high ratio of AL/BC in an emmetropic eye is a risk factor for the development of myopia, where the resultant myopia is due to increased AL or corneal power [1].

A flatter corneal curvature and a decrease in CCT have been correlated with greater AL in the myopic eye [5]. Eyes with a higher level of myopia are associated with lesser flattening of the cornea (steeper cornea) and greater AL. Contradicting studies have thus correlated steeper corneas with increasing AL [6]. The mean CCT in our study was 533.87 microns (SD 40.02). The values of CCT had a normal distribution. This value is similar to that shown in a study in Taiwan but differs by 10 microns from the mean values reported in studies done in North India and Singapore [5,7-8]. The normal value of CCT in healthy adults varies from 507 to 565 microns [7].

The CCT had a negative correlation with SE (r= -0.119; p=0.14). There was a positive correlation between CCT with AL (r= 0.211; p=0.008) in this study. This shows that axial myopes tend to have thicker corneas. Some studies have stated that there is no significant correlation between CCT and AL [8-9].

The present study has highlighted the correlation between corneal curvature and CCT, indicating that steeper corneas tend to be thinner (r=0.269; p=0.001). A study done in Singaporean school children and a study in children with Down’s syndrome have demonstrated a similar finding [8,10]. Eyes with keratoconus also have very steep and thin corneas. It has been suggested that a thin cornea is more prone to yield to the intraocular pressure and tends to become steeper [10]. An Indian study has reported that there was no correlation between CCT and curvature [7]. The discrepancy in the findings may be due to the difference in the method used for estimating CCT. While an ultrasonic pachymeter was used in our study, the other study had utilized an optical slit lamp-mounted pachymeter. This may partly explain the observed variation.

## Conclusions

SE of refraction correlates well with AL. Increase in the corneal power is associated with increasing myopia. Increasing corneal curvature (steeper cornea) is correlated well with increasing myopic refraction and thinner cornea. A longer AL is associated with thicker cornea or flatter cornea. These ocular biometric findings have crucial implications in refractive surgeries.
